# Return to Play after SARS-CoV-2 Infection in Competitive Athletes of Distinct Sport Disciplines in Italy: A FMSI (Italian Federation of Sports Medicine) Study

**DOI:** 10.3390/jcdd9020059

**Published:** 2022-02-15

**Authors:** Maurizio Casasco, Ferdinando Iellamo, Marco Scorcu, Attilio Parisi, Irena Tavcar, Erica Brugin, Barbara Martini, Chiara Fossati, Fabio Pigozzi

**Affiliations:** 1 Italian Federation of Sports Medicine (FMSI), 00196 Rome, Italy; presidente@fmsi.it (M.C.); mscorcu@tiscali.it (M.S.); attilio.parisi@uniroma4.it (A.P.); irenat211@gmail.com (I.T.); ericabrugin81@yahoo.it (E.B.); chiara.fossati@uniroma4.it (C.F.); fabio.pigozzi@uniroma4.it (F.P.); 2 Post-Graduate School of Sport Medicine and Physical Exercise, University Tor Vergata, 00173 Rome, Italy; 3 Istituto di Ricovero e Cura a Carattere Scientifico San Raffaele Pisana, 00163 Roma, Italy; 4 Sports and Exercise Medicine Service, ATS Sardegna, 09123 Cagliari, Italy; 5 Department of Movement, Human and Health Sciences, University “Foro Italico”, 00135 Rome, Italy; 6 Cardiovascular Rehabilitation and Sports Medicine Service, 30033 Noale, Italy; 7 Department of Enterprise Engineering, University Tor Vergata, 00173 Rome, Italy; barbara.martini@uniroma2.it

**Keywords:** COVID-19 infection, athletes, return to play

## Abstract

Background: SARS-CoV-2 can lead to several systemic complications, including myocardial injuries; these might be worsened by heavy physical activity. The optimal approach to cardiac risk stratification following SARS-CoV-2 infection in athletes for a safe return to play (RTP) still needs defining. The aim of this study was to assess the prevalence of abnormal RTP test results, according to the protocol of Italian Federation of Sport Medicine (FMSI), which was endorsed by the Italian Ministry of Health, potentially representing COVID-19-associated cardiac injuries. Methods: This was a prospective, multicenter, observational study. All consecutive competitive athletes who underwent COVID-19 RTP testing protocol from 1 May to 31 July 2021, across 60 Italian Centers of Sports Medicine, were enrolled in the study. Athletes were tested at least 30 days after negativization of the nasopharyngeal swab (or immediately after negativization in professional athletes or Probable Olympians). A 12-lead electrocardiography at rest and during maximal incremental exercise test with continuous O_2_ saturation monitoring and an echocardiographic examination were part of the protocol. In athletes with “moderate” disease (NHI classification), 24 h ECG monitoring (to be performed on a training day) and Magnetic Resonance Imaging (MRI) were also performed. Results: A total of 4143 athletes (67.8% males and 32.2% females) (53% > 18 years, 20% 18–35 years and 16% > 35 years), from more than 40 different sport disciplines, were included in the study. The mean age was 22.5 ± 13.3 years, with ages ranging from 8 to 80 years. Of these athletes, 52.3% were asymptomatic, 46.4% manifested mild symptoms, 1.1% and 0.14% had moderate or severe symptoms, respectively, while critical illness was evident in one athlete. Abnormal echocardiographic findings were detected in 80 cases (1.9%), and pericarditis in 7 cases (0.2%); all were from mildly symptomatic athletes. Arrhythmic events were recorded in 239 athletes, with 224 (5.4%) in the exercise test and 15 (0.4%) during 24 h ECG monitoring. Ventricular arrhythmias were observed in 101 (2.4%) athletes from the total population (mostly isolated or couples of premature ventricular beats): 91 in the exercise test and 10 during 24 h ECG monitoring. Cardiac magnetic resonance was performed in 34 athletes; the presence of myocarditis was confirmed in 5 athletes (0.12% of the total population, 14.7% of athletes in which MRI was performed). Conclusions: According to our results, cardiac complications from SARS-CoV-2 in asymptomatic or mildly symptomatic competitive athletes are rare, and an RTP assessment based on symptoms and ECG-monitored exercise test would ensure a safe RTP in these athletes.

## 1. Introduction

Return to play (RTP) for competitive athletes infected with SARS-CoV-2 who recovered from the disease is a matter of great social interest and of health policy. In fact, particular concerns have been raised regarding possible adverse cardiac sequelae of SARS-CoV-2 infection [1] However, limited data are available regarding the prevalence of cardiac injury and its consequences among non-hospitalized individuals with SARS-CoV-2, an issue highlighted by the young age of athletes and the large absence or, at most, the presence of mild clinical symptoms. 

Infection from the SARS-CoV-2 virus might result in myocardial injury, which can be worsened by physical efforts, particularly in the acute phase when viral replication can be enhanced by vigorous physical activity, resulting in greater structural damage of the heart, particularly myocarditis. Myocarditis could then result in cardiac dysfunction, clinically relevant arrhythmias, or even death. However, the prevalence of cardiac injury in non-hospitalized athletes is not well defined, and neither are the post-infection durations and the magnitude of possible complications. 

Currently, a cardiac injury from COVID-19 is defined as having high-sensitivity troponin I (hsTn) levels, higher than the 99th percentile of the laboratory, electrocardiographic and/or echocardiographic abnormalities (including pericarditis), or Magnetic Resonance Imaging (MRI).

The optimal approach to cardiac risk stratification for athletes’ RTP after SARS-CoV-2 infection is still to be defined. This may cause, and might have resulted in, an excessive utilization of diagnostic procedures of unproven utility for the individuation of cardiac injury from SARS-CoV-2 infection and subsequent RTP, with an elevated burden, even economic, on the public health care system. At the beginning of the pandemic, a prudential approach could have been justified by lack of knowledge about the disease and its evolution. However, with advances of knowledge acquired from several studies, the protocols for RTP could be reconsidered and revised.

A number of studies have aimed to determine cardiac risk stratification in athletes over the last two years of the pandemic [2,3,4,5,6,7,8]. However, the relatively small sample sizes of the studies, the single-centered approach, the small number of sport disciplines assessed and the underrepresentation of female athletes limit definitive conclusions and the generalizability of the results obtained to date.

Therefore, large studies around the world on athletes from all sport disciplines, which take into account gender (and race) differences, and data collection on the current pandemic appear mandatory.

Accordingly, this prospective, observational study, sponsored by the Italian Federation of Sports Medicine (FMSI), was conceived in order to collect systematic cardiac RTP COVID-19 testing results. It would provide data on the prevalence of clinically detectable and relevant cardiac injury in a large number (over 4000) of athletes of both sexes, practicing more than 40 distinct sport disciplines, who tested positive for COVID-19. We aimed to verify the value of our approach in detecting cardiac injuries for achieving a safe return to competitive sports.

## 2. Methods

Sixty-one Centers of Sports Medicine distributed across Italy enrolled all consecutive athletes who underwent COVID-19 RTP protocol during the study time (1 May to 31 July 2021), after having tested positive for COVID-19 infection. 

SARS-CoV-2 infection was diagnosed by polymerase chain reaction testing. The evaluation screening was performed at least 30 days after negativization of the nasopharyngeal swab (or immediately after negativization in professional athletes or Probable Olympians).

According to NIH classification, athletes were divided in three classes: Class A1 included asymptomatic/pauci-symptomatic athletes and those with “mild” disease; Class A2 included athletes with “moderate” disease or those needing hospitalization; Class A3 athletes had “severe” and/or “critical” disease.

The components of the cardiac screening protocol for RTP in Class A1 athletes, as dictated by the Italian Ministry of Health, based on the proposal set up by the FMSI [9], included a 12-lead ECG at rest and during maximal incremental exercise testing with continuous O_2_ saturation monitoring and an echocardiographic examination.

In Class A2 athletes, a 24 h ECG monitoring (to be performed on a training day) and respiratory function assessment were added to the above diagnostic tests.

The sports medicine physicians evaluated the need for additional testing on a case-by-case basis. These included: pulmonary imaging; lung diffusion capacity for carbon monoxide (DLCO); cardiopulmonary exercise test (CPET); MRI.

The following blood chemistry assessments were also performed: blood count; ALT/AST; gamma GT; creatinine; CPK; hs-c-TnT; LDH; PT/PTT; INR; protein electrophoresis; PCR; D-dimer; Ferritin.

In Class A3 athletes, a CPET was mandatory, in addition to the above tests. 

Professional athletes were rated as Class A3 non-professional athletes, regardless of the severity of the disease. 

Abnormal ECG results were defined according to international recommendations [10]. Echocardiographic findings raising concern for potential cardiac injury were determined by examining physicians. To facilitate the collection and transmission of data, a web form was used on the FMSI institutional website, which was accessed following authentication with a Username and Password to ensure the protection of privacy.

The study protocol was approved by the institutional review board of the FMSI. Patient characteristics, SARS-CoV-2 symptoms, and cardiac testing results were recorded using basic descriptive methods, including frequency distributions, means, and standard deviations. This study followed the Strengthening the Reporting of Observational Studies in Epidemiology (STROBE) reporting guidelines for cross-sectional studies. As this was a descriptive study, comparisons between groups and statistical analyses were not performed [3].

## 3. Results

A total of 4143 athletes were included in the study: 2810 (67.8%) were males and 1333 (32.2%) were females. Of these, 2235 (53.9%) athletes were <18 years old, 1221 (29.5%) were between 18 and 35 years old and 687 (16.6%) were >35 years old. The mean age was 22.5 ± 13.3 years, with ages ranging from 8 to 80 years. Athletes practiced more than 40 different sport disciplines (Table 1).

**Figure 1:**
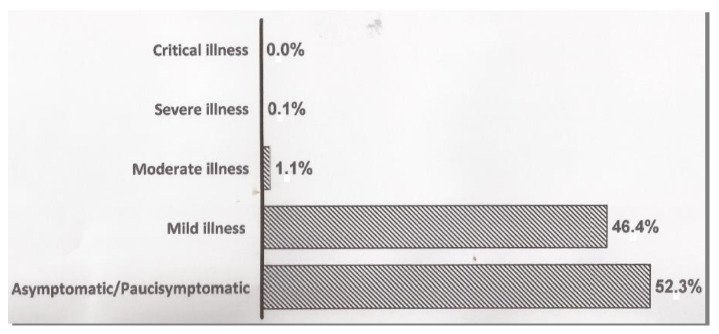
Clinical spectrum of SARS-CoV-2 infection.

Asymptomatic infection occurred in 2168 athletes (52.3%), whereas mild symptoms had been manifested in 1924 (46.4%) athletes. Moderate and severe COVID-19 infection occurred in 44 (1.1%) and 6 (0.14%) athletes, respectively. Only one subject (0.02%) showed critical illness (Figure 1). Mild symptoms have been defined as nonspecific and self-limited fatigue, non-persistent fever, anosmia or ageusia, nausea, vomiting, diarrhea, headache, cough without dyspnea, sore throat and nasopharyngeal congestion [9,11].

The vast majority of asymptomatic and/or pauci-symptomatic athletes (~60%) were less than 18 years old. More than 92% of athletes had no previous history of cardiac diseases. A history of cardiac disease did not influence the clinical presentation (97% vs. 99% with no- or mild symptoms, respectively).

Abnormal echocardiographic findings were detected in 80 cases (1.9%). Among those, 16 athletes developed arrhythmias at the ergometric test, with 5 cases of premature ventricular beats (PVBs).

Only 7 cases (0.2%) of pericarditis were detected (not including the presence of detachment of the pericardial leaflets), all of which occurred in mildly symptomatic athletes who returned to play in less than 30 days (3 athletes) or between 30 and 60 days.

Overall, 224 athletes (5.4%) showed arrhythmic events at ergometric testing, with 27 (12%) having a known pre-infection history compatible with cardiac damage-inducible arrhythmias and 15 athletes (6.70%) with known pre-existing (i.e., pre-COVID) PVBs. Another 15 athletes (0.36%) showed arrhythmic events during 24 h ECG monitoring.

**Figure 2:**
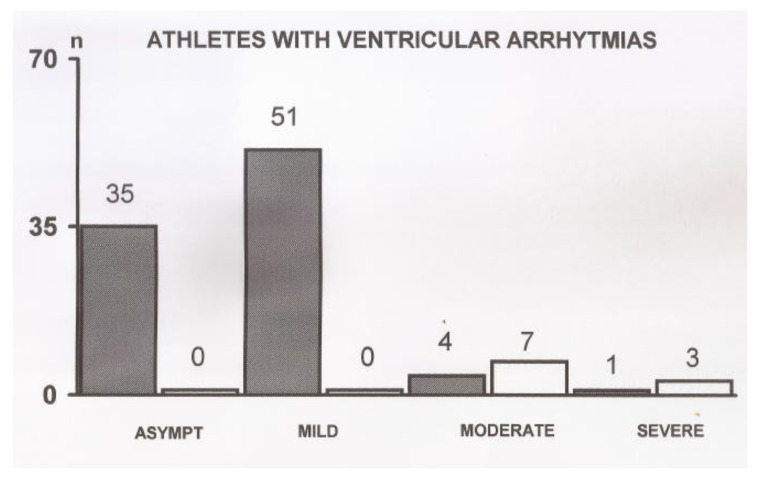
Occurrence of premature ventricular beats according to the severity of clinical presentation. Asympt = asymptomatic and pauci-symptomatic athletes. Hatched bars indicate arrhythmias detected at the ergometric test; white bars indicate arrhythmias detected during 24 h ECG monitoring.

Ventricular arrhythmias were observed in 91 (2.20%) athletes and consisted mostly of isolated PVBs and couples of PVBs. Among these, there were 7 cases of bigeminy, 3 cases of trigeminy and 12 cases of PVBs couples. In one athlete, a run of PVBs occurred during exercise, not accompanied by alarm symptoms. Arrhythmic events are described in detail in Table 2. The occurrence of PVBs, according to the severity of clinical presentation, is shown in Figure 2. Another 10 athletes (0.24%) showed arrhythmic events during 24 h ECG monitoring.

In athletes with arrhythmias, RTP was more delayed in comparison to athletes having cardiac abnormalities, but without arrhythmias.

Cardiac magnetic resonance (CMR) imaging was performed in 34 athletes (0.8%), mainly because of the occurrence of arrhythmias at exercise testing or abnormalities at echocardiography. CMR imaging confirmed myocarditis in 5 out of these 34 athletes (representing 0.12% of the overall population and 14.7% of athletes in which MRI was performed).

## 4. Discussion

The main findings of this prospective survey study indicate that cardiac complications attributable to SARS-CoV-2 infection are rare in competitive athletes, and RTP screening based on symptoms, resting ECG and ECG-monitored exercise testing is capable of detecting cardiovascular complications following SARS-CoV-2 infection and could permit a safe RTP.

To our knowledge, this is one of the few studies to include exercise testing among routine screening for RTP in a large sample of competitive athletes. This allowed us to discover that arrhythmias are the more frequent cardiac alterations in athletes recovering from SARS-CoV-2 infection. In these athletes, RTP was also more delayed in comparison to those with cardiac abnormalities but without arrhythmias. Surprisingly, few small studies [6] included ET as mandatory in the screening for RTP after SARS-CoV-2 infection, despite it being well known that effort may unmask arrhythmias not present at rest [11]. Additionally, exercise stress testing (e.g., step test) is a fundamental component of the annual pre-participating screening in competitive and professional athletes in Italy, according to FMSI rules approved by the Italian law. In this context, it is worth mentioning that the FMSI is the only scientific society recognized by the Ministry of Health in the field of Sports Medicine in Italy.

In our survey study, pericarditis was only detected in 7 athletes from the overall population. This finding is in line with most, though not all [12] RTP studies performed so far.

Previous studies on RTP in athletes after COVID-19 infection were conducted using small sample sizes, often in athletes from a single sport discipline and using males only. These limitations prevented the generalizability of results. Our study was conducted using a large sample size, with athletes of both sexes, who were practicing over 40 distinct sport disciplines (Table 2), spread throughout Italy, allowing greater generalization of results in comparison to previous studies. Overall, our results confirmed, and extended, to a large population where previous results obtained in small, mainly single-centered studies could not, that cardiac complications in competitive athletes who tested positive for COVID-19 infection are rare (although not to be underestimated).

The findings from our survey study give robust support to expert consensus ACC recommendations [13] which do not advocate for cardiovascular risk stratification in athletes who were asymptomatic or with mild SARS-CoV-19 and remain asymptomatic after completion of appropriate self-isolation [2]. 

*Limitations*. Cardiac testing of our RTP protocol did not include routine CMR. This choice was dictated by several concerns regarding using CMR imaging as a screening tool. CMR imaging might not be easily accessible to all local Sport Medicine and Clinical Cardiology Centers, as there is a lack of expertise in interpretation, leading to inter-reader variability, the paucity of normative CMR data in athletes and, finally, considering the cost-effectiveness of this diagnostic tool without a net clinical benefit [14]. Our reasoning was also based on the findings of many studies reporting that CMR imaging confirmed a diagnosis of a suspected inflammatory heart disease or frank myocarditis in a very small percentage of athletes undergoing this diagnostic tool (0.6% and 0.4% of the total cohorts studied, respectively). These small percentages have been reported in the vast majority of studies, although few studies reported higher rates of COVID-19 myocarditis and pericarditis in small cohorts of athletes [15,16]. To add uncertainty, there is no uniformly accepted definition of cardiac involvement attributable to SARS-CoV-2 infection.

Hence, from the present study, and other studies, we can conclude that CMR imaging should be reserved for selected athletes with protracted cardiac symptoms or new, pathologic findings from other diagnostic tools. Additionally, 24 h ECG monitoring was not performed on mildly symptomatic patients. This was in agreement with the decree issued by the Italian Ministry of Health, based on the proposal set up by the FMSI [9].

Finally, our study did not report outcomes and long-term prognostic data on cardiac complications found after SARS-CoV-2. This information is still lacking in athletes (and in the general population as well), likely reflecting the relatively recent discovery of COVID-19-related illness. It should also be mentioned that the multi-center nature of this study, with possible differences among centers in the interpretation of data, might have affected our estimates.

Analogue concerns apply to troponin assessment, which was performed as a routine test only in Class A2 and Class A3 athletes. Even though troponin is included in some screening protocols for RTP, it has rarely been found positive, even in athletes with SARS-CoV-19 infection [3,4,7,16], and not confirmed by CMR imaging [8].

These data support recommendations that biomarkers of cardiac injury should be interpreted in the context of clinical manifestations and cardiac test results and are not indispensable within a routine RTP protocol [16].

## 5. Conclusions

The results of this study, obtained using a large population of athletes of both sexes who were practicing numerous, distinct sport disciplines, indicate that cardiac complications from SARS-CoV-2 in asymptomatic or mildly symptomatic competitive athletes are rare, and that an RTP assessment based on symptoms and ECG-monitored exercise testing would be able to ensure a safe RTP. This would fulfill the need to balance resource management across health care systems, which are essential elements to incorporate in the refinement of RTP screening practices.

## Figures and Tables

**Table 1:** Number of athletes in the different sport disciplines *.

	Males				Females				
	Under 18	18–35	Over 35	Overall Males	Under 18	18–35	Over 35	Overall Females	Overall Athletes
SOCCER	631	342	43	1016	41	29	3	73	1089
BASKETBALL	282	107	7	396	42	17	0	59	455
VOLLEY	52	32	10	94	264	92	4	360	454
ATHLETICS	57	72	113	242	40	36	34	110	352
GYMNASTICS	13	15	6	34	166	18	8	192	226
TENNIS	30	36	80	146	15	5	28	48	194
SWIMMING	54	15	24	93	60	15	15	90	183
CYCLING	21	35	89	145	1	2	6	9	154
SPORT DANCE	13	5	8	26	63	19	10	92	118
RUGBY	40	21	2	63	4	4	1	9	72
FIGURE SKATING	8	1	0	9	53	8	1	62	71
BOXING	8	26	6	40	2	1	1	4	44
ICE HOCKEY	17	20	3	40	1	1	0	2	42
TRIATHLON	0	9	27	36	0	2	2	4	40
WATER POLO	22	11	1	34	1	2	0	3	37
WEIGHTLIFTING	2	13	12	27	1	4	4	9	36
BASEBALL	20	9	3	32	1	0	0	1	33
EQUESTRIAN SPORTS	0	1	1	2	21	8	1	30	32
ALPINE SKIING	5	10	7	22	6	2	2	10	32
KARATE	8	9	4	21	4	2	0	6	27
MOTORCYCLING	5	12	9	26	0	0	1	1	27
KICK BOXING	5	8	5	18	2	3	0	5	23,
JUDO	7	8	2	17	2	3	0	5	22
ROWING	5	2	4	11	3	2	2	7	18
FENCING	9	2	1	12	4	2	0	6	18
SPORT CLIMBING	4	3	3	10	5	0	1	6	16
TAEKWON-DO	6	3	3	12	3	0	1	4	16
CANOEING/KAYAK	4	5	1	10	4	0	1	5	15
GOLF	1	2	9	12	0	0	1	1	13
FIELD HOCKEY	3	2	0	5	3	4	1	8	13
SYNCHRONIZED SWIMMING	0	0	0	0	9	2	0	11	11
SPEED SKATING	3	1	0	4	6	1	0	7	11
SOFTBALL	0	0	0	0	8	3	0	11	11
ICE FIGURE SKATING	0	1	0	1	7	2	0	9	10
TABLE TENNIS	3	2	2	7	2	0	1	3	10

* Only sport disciplines with at least 10 athletes are reported.

**Table 2:** Details of arrhythmic events.

	Males				Females			
**Age**	<18	18–35	>35	sub-set	<18	18–35	>35	sub-set
**Nr of athletes with Arrhythmias**	42	68	79	189	10	20	20	50
% on the cohort	17.6%	28.5%	33.1%	79.1%	4.2%	8.4%	8.4%	20.9%
								
Of which with pre-infection history	0	5	14	19	1	1	7	9
% on the cohort	0%	2.1%	5.9%	7.9%	0.4%	0.4%	2.9%	3.8%
								
	**Supraventricular premature beats**							
	**Males**				**Females**			
	<18	18–35	>35	sub-set	<18	18–35	>35	sub-set
**Nr of athletes with SPBs**	21	27	43	91	3	8	6	17
% on the cohort	19.4%	25.0%	39.8%	84.3%	2.8%	7.4%	5.5%	15.7%
								
Of which with pre-infection history	0	1	8	9	0	1	2	3
% on the cohort	0 %	0.9%	7.4%	8.3%	0 %	0.9%	1.8%	2.8%
								
	**Premature ventricular beats**							
	**Males**				**Females**			
**Age**	<18	18–35	>35	sub-set	<18	18–35	>35	sub-set
**Nr of athletes with PVBs**	12	35	34	81	3	8	9	20
% on the cohort	11.9%	34.7%	33.7%	80.2%	2.9%	7.9%	8.9%	19.8%
Of which with pre-infection history	0	3	8	11	1	0	2	3
% on the cohort	0 %	2.9%	7.9%	10.9%	0.9%	0%	1.9%	2.9%
								
**Complexity**								
Isolated	9	28	27	64	3	3	6	12
	8.9%	27.7%	26.7%	63.4%	2.9%	2.9%	5.9%	11.9%
Bigeminy	1	1	1	3	0	2	2	4
	0.9%	0.9%	0.9%	2.9%	0%	1.9%	1.9%	3.9%
Trigeminy	1	0	1	2	0	1	0	1
	0.9%	0%	0.9%	1.9%	0%	0.9%	0%	0.9%
Couples	0	5	5	10	0	2	1	3
	0 %	4.9%	4.9%	9,9%	0%	1.9%	0.9%	2.9%
Runs	1	1		2				0
	0.9%	0.9%	0.00%	1.9%	0%	0%	0%	0%
